# Birth characteristics and childhood carcinomas

**DOI:** 10.1038/bjc.2011.359

**Published:** 2011-09-13

**Authors:** K J Johnson, S E Carozza, E J Chow, E E Fox, S Horel, C C McLaughlin, B A Mueller, S E Puumala, P Reynolds, J Von Behren, L G Spector

**Affiliations:** 1 The Brown School and Department of Pediatrics, Washington University in St Louis, St Louis, MO 63130, USA; 2 Department of Public Health, Oregon State University, Corvallis, OR 97331, USA; 3 Public Health Sciences Division, Fred Hutchinson Cancer Research Center, Seattle, WA 98109, USA; 4 Department of Pediatrics, University of Washington, Seattle, WA 98195, USA; 5 Center for Clinical and Translational Sciences, University of Texas Health Science Center, Houston, TX 77225, USA; 6 Patient Safety Center, New York State Department of Health, Albany, NY 12237, USA; 7 Department of Epidemiology, University of Washington, Seattle, WA 98195, USA; 8 Methodology and Data Analysis Center, Sanford Research, Sioux Falls, SD 57104, USA; 9 Department of Pediatrics, Sanford School of Medicine, University of South Dakota, Sioux Falls, SD 57104, USA; 10 Northern California Cancer Center, Berkeley, CA 94538, USA; 11 Division of Epidemiology/Clinical Research, Department of Pediatrics, University of Minnesota, Minneapolis, MN 55455, USA; 12 University of Minnesota Masonic Cancer Center, Minneapolis, MN 55455, USA

**Keywords:** paediatric, carcinoma, melanoma, thyroid, risk

## Abstract

**Background:**

Carcinomas in children are rare and have not been well studied.

**Methods:**

We conducted a population-based case–control study and examined associations between birth characteristics and childhood carcinomas diagnosed from 28 days to 14 years during 1980–2004 using pooled data from five states (NY, WA, MN, TX, and CA) that linked their birth and cancer registries. The pooled data set contained 57 966 controls and 475 carcinoma cases, including 159 thyroid and 126 malignant melanoma cases. We used unconditional logistic regression to calculate odds ratios (ORs) and 95% confidence intervals (CIs).

**Results:**

White compared with ‘other’ race was positively associated with melanoma (OR=3.22, 95% CI 1.33–8.33). Older maternal age increased the risk for melanoma (OR_per 5-year age increase_=1.20, 95% CI 1.00–1.44), whereas paternal age increased the risk for any carcinoma (OR=1.10_per 5-year age increase_, 95% CI 1.01–1.20) and thyroid carcinoma (OR_per 5-year age increase_=1.16, 95% CI 1.01–1.33). Gestational age <37 *vs* 37–42 weeks increased the risk for thyroid carcinoma (OR=1.87, 95% CI 1.07–3.27). Plurality, birth weight, and birth order were not significantly associated with childhood carcinomas.

**Conclusion:**

This exploratory study indicates that some birth characteristics including older parental age and low gestational age may be related to childhood carcinoma aetiology.

In contrast to adults, carcinomas in children are extremely rare with an estimated annual incidence among those 0–14 years of age of 6.3 cases per million (Surveillance, Epidemiology, and End Results, 2008). The incidence of childhood carcinomas increases with age with most cases being diagnosed after age 14 years. The major childhood carcinoma subtypes are renal, hepatic, gonadal, adrenocortical, thyroid, nasopharyngeal, and malignant melanoma (Steliarova-Foucher *et al*, 2005). Thyroid carcinoma and melanoma comprise 60% of cases (Surveillance, Epidemiology, and End Results, 2008). As is the case for adults, there is a female predominance for both thyroid carcinoma and malignant melanoma (Pizzo and Poplack, 2006). Both of these subtypes are more common in whites than blacks. The incidence of paediatric malignant melanoma has increased over recent decades for unknown reasons. Notably, from 1992 to 2007, the incidence of paediatric malignant melanoma has increased significantly at an average rate of 4.8% per year (Surveillance, Epidemiology, and End Results, 2008).

The current understanding of the aetiology of the two most common paediatric carcinomas, thyroid carcinoma and malignant melanoma, is predominantly based on case-series reports and studies of rare syndromes. Syndromes associated with thyroid carcinomas include the following: familial adenomatous polyposis, multiple endocrine neoplasia type 2, Cowden syndrome, Carney complex, Pendred syndrome, and Werner syndrome (Richards, 2010). Xeroderma pigmentosum, a DNA repair disorder, is associated with an increased risk of paediatric melanoma (Pizzo and Poplack, 2006). Germline mutations in *CDKN2A* and *CDK4* (Udayakumar and Tsao, 2009) have been linked to familial melanoma, however mutations in both of these genes have been infrequently reported in young onset melanoma (Tsao *et al*, 2000; Berg *et al*, 2004; Debniak *et al*, 2008). Other biological risk factors for childhood melanoma include giant congenital melanocytic nevi, familial dysplastic nevus syndrome, fair skin, light hair colour, blue or green eyes, immunosuppression, and atypical or numerous nevi (Jen *et al*, 2009). Ionising radiation exposure is an established risk factor for adult and childhood thyroid carcinoma (Ron, 2007). Numerous adult melanoma studies have noted an increased risk in relation to sun exposure (Schottenfeld and Fraumeni, 2006), however the extent to which this is associated with childhood melanoma is unclear (Whiteman *et al*, 1997; Youl *et al*, 2011).

Early life is thought to be critical for cancer development in children (Boice and Miller, 1999; Greaves, 2003). To our knowledge, no studies have specifically examined the association between birth characteristics and childhood carcinomas. Using data from a registry-based linkage study, our objective was to conduct an exploratory analysis of associations between factors recorded in birth records and childhood carcinomas, both overall and separately for the two major subtypes (thyroid carcinoma and malignant melanoma). Because of the size and scope of this study, it may offer additional clues to the aetiology of carcinomas in children.

## Materials and Methods

Study procedures have been reported in detail previously (Johnson *et al*, 2009; Puumala *et al*, 2009; Spector *et al*, 2009; Carozza *et al*, 2010; Chow *et al*, 2010). Institutional Review Board approvals were obtained from each state's health department and participating institutions before conducting the study. Individuals who were diagnosed with childhood cancer during 1980–2004 were identified through the population-based central cancer registries of California, Minnesota, New York (excluding New York City), Texas, and Washington states. Briefly, childhood cancer cases in each state's cancer registry were linked to their respective birth certificates using sequential deterministic or probabilistic record linkage (Jaro, 1995). Cases were classified according to the International Classification of Childhood Cancer 3rd edition (Steliarova-Foucher *et al*, 2005). All cases of melanoma were malignant. Age at diagnosis in months was obtained from cancer registry records. Controls were randomly selected from each state's birth registry in ratios to cases that varied from 1 to 10. Frequency matching was used in four states and individual matching in one (CA); year of birth was a matching factor in all states although two also matched on sex (CA, TX).

Additional criteria were applied before pooling to ensure uniformity of data. Cases that were also selected as controls in the case-cohort sampling strategy employed by Minnesota and New York were excluded from the control group in this analysis. California cases included those diagnosed at <28 days old, whom we subsequently excluded for consistency with other states. Subjects with reported Down syndrome (*n*=100) were excluded, although it should be noted that Down syndrome was not recorded in Texas before 1984 or in Washington before 1989. The final pooled data set included 17 672 cases and 57 966 controls.

### Statistical analyses

Odds ratios (ORs) and 95% confidence intervals (CIs) were calculated by unconditional logistic regression (SAS version 9.1, SAS, Cary, NC, USA); individual matching in the California data set was broken for this reason. Race was categorised as white and non-white using maternal race as a proxy for the child's race. We modelled associations with parental age both as continuous variables in 5-year increments and as categorical variables (<25, 25–29, 30–34, and ⩾35 years). Categorical variables were used to assess the appropriateness of the log linear model for associations between parental age and childhood carcinomas. Each parent's age was examined in separate models and in models that included maternal and paternal age variables. Gestational age was categorised as preterm (<37 weeks), term (37–42 weeks), and post-term (>42 weeks). Birth weight was modelled as a continuous variable in 500-g increments and in categories using the following cutoffs for low (<2500 g), normal (2500–4000 g), and high birth weight (>4000 g). All models were adjusted for state and the matching variables sex and birth year. Because knowledge about risk factors for childhood carcinomas is sparse we included all available potential confounders of associations between birth characteristic and childhood carcinoma. Therefore, in addition to state, birth year, and sex, models included gestational age, plurality, birth weight, birth order and maternal or paternal age. Effect modification on the multiplicative scale was assessed by including the cross product of the variables of interest in the logistic regression model. We included only subjects with complete data on all variables. The percentage of cases and controls with missing data on the variables included in this analysis was similar for sex (0% *vs* 0.02%), maternal age (0% *vs* 0.04%), paternal age (11% *vs* 11%), plurality (0% *vs* 0.6%), birth weight (0% *vs* 0.6%), gestational age (6.1% *vs* 4.6%), birth order (3.4% *vs* 2.6%), and maternal race (3.2% *vs* 1.5%). The number of subjects that were excluded from each model is detailed in the table 2 footnote. ORs were considered statistically significant if the *P*-value was ⩽0.05.

## Results

A total of 475 children diagnosed with carcinomas between 1980 and 2004 were identified in the pooled data set (Table 1). Thyroid carcinomas (*n*=159) were most common followed by melanomas (*n*=126), and together constituted more than half (60%) of the carcinomas in this study. The median diagnosis age was 11.4 years for carcinomas overall (data not shown) with most cases being diagnosed between 10 and 14 years overall and for most subtypes. More females were diagnosed with carcinomas than males particularly for thyroid carcinomas. By race, there were notable differences for melanoma where very few cases were non-white. This is in contrast to renal cell carcinomas and nasopharyngeal carcinomas where a larger percentage were non-white, 44% and 38%, respectively, compared with controls (14.7% (data not shown)). Because of the small number of cases for most cancer sites, associations with birth characteristics are presented for all carcinomas combined and for the two sites with 100 or more cases (malignant melanoma and thyroid carcinoma).

Maternal white race compared with ‘other’ race conferred a significantly increased risk for melanoma (OR=3.27, 95% CI 1.33–8.06) but not for all carcinomas or thyroid carcinoma (Table 2).

Maternal age modelled as a continuous variable was associated with a marginally significant increased risk for childhood carcinomas overall (OR_per 5-year increase_=1.09, 95% CI 0.99–1.20). Both thyroid carcinoma and melanoma showed a positive linear maternal age effect that was significant for melanoma (OR_per 5-year increase_=1.20, 95% CI 1.00–1.44). In models that categorised maternal age, there was a consistent nonsignificant pattern of increasing risk with advancing maternal age category for all carcinomas. However, there was some evidence for an association of older maternal age for malignant melanoma where all maternal age categories were associated with elevated risks compared with the reference group and for thyroid carcinoma in the offspring of women born to mothers ⩾35 *vs* <25 years (OR=1.87, 95% CI 1.01–3.48). There was no significant effect modification by birth year category (1975–1984 *vs* 1985–2004) between maternal age modelled as a continuous variable and all carcinomas, thyroid carcinomas, or malignant melanomas.

Paternal age modelled as a continuous variable was associated with a significant positive linear trend for childhood carcinomas overall (OR_per 5-year increase_=1.10, 95% CI 1.01–1.20) and thyroid carcinoma (OR_per 5-year increase_=1.16, 95% CI 1.01–1.33) and a nonsignificant positive linear trend for malignant melanoma (OR=1.13, 95% CI 0.96–1.32). In models where paternal age was modelled categorically, the risk for all childhood carcinomas and thyroid carcinomas specifically generally increased with increasing paternal age, consistent with the linear model of paternal age; however all estimates were imprecise. There was no significant effect modification by birth year category (1975–1984 *vs* 1985–2004) between paternal age modelled as a continuous variable and all carcinomas, thyroid carcinomas, or malignant melanomas. In models that included maternal and paternal age, the independent effects of each were attenuated (data not shown).

Plurality, birth weight, and birth order were not significantly associated with all carcinomas, thyroid carcinoma, or melanoma. Gestational age <37 weeks *vs* 37–42 weeks was associated with a significant increased risk of thyroid carcinoma (OR=1.87, 95% CI 1.07–3.27). Also of note is that the risk for melanoma appeared to decrease in relation to increasing birth order category, albeit CIs were wide; for birth orders of four or higher *vs* first born the OR was 0.49 (95% CI 0.21–1.12).

We also explored whether associations between parental age and childhood carcinomas varied by age of diagnosis and sex (Supplementary Table 1). The risk estimates for the association between parental age and childhood carcinoma for those diagnosed ⩽11 years of age *vs* >11 years of age were generally similar. However, we note that there was a consistently stronger parental age association for individuals in the younger *vs* older age category. Associations between parental age and carcinomas between sexes were not statistically significantly different.

## Discussion

In addition to the previously documented risk factor for melanoma of white race, (Schottenfeld and Fraumeni, 2006) we observed a weak parental age effect for both thyroid carcinomas and melanomas. For thyroid carcinomas, the data suggest a paternal age effect, whereas for melanomas the data support a maternal age effect. We also observed an increased risk of thyroid carcinoma in association with gestational age <37 weeks and a decreased risk of melanoma with increasing birth order.

Older parental age has been associated with a modest risk increase for several of the more common childhood cancers including leukaemia, central nervous system tumours, and Wilm's tumour in some but not all of the larger studies (Dockerty *et al*, 2001; Yip *et al*, 2006; Johnson *et al*, 2009; Schuz *et al*, 2011). For malignant melanoma diagnosed at any age, no increased risk was found in association with maternal or paternal age in a study of all Norwegian cases (*n*=709) born between 1967 and 1986 followed until 31 December 2003 (Franco-Lie *et al*, 2008). A significant positive trend with increasing maternal age at first birth but not birth of the index child was found in a study of 2 594 783 Danes that included 1674 incident malignant melanoma cases followed from 1968 to 2002 who were born between 1950 and 2002 (Olesen *et al*, 2009). Although it involved older cases than those in our study, a Swedish study reported a significant 30% excess risk of malignant melanoma in the offspring (aged 15–53 years) of mothers who were >40 years compared with those <20 years at their time of birth, whereas a 28% decreased risk was reported in the offspring of fathers who were >40 *vs* <25 at the time of their birth. No significant associations were found between maternal or paternal age and thyroid cancer (Hemminki and Kyyronen, 1999), while another study also found a protective effect of paternal age on thyroid carcinoma risk (Galanti *et al*, 1997). The vast majority of individuals in these studies were >15 years at the time of their cancer diagnosis.

Several hypotheses have been proposed to explain the increased risks of various diseases in offspring in relation to advanced parental age. The most plausible explanation for a paternal age effect is the accumulation of *de novo* mutations that are thought to occur in aging gametes that divide throughout the male lifetime (Crow, 2000). It is possible that a paternal age effect for young onset carcinomas could be mediated by *de novo* mutations in the paternal germline in genomic regions that predispose to carcinomas. The accumulation of mutations as an underlying explanation for a maternal age effect is less likely because oocyte cell division is completed before birth. A maternal age effect is well known for Down syndrome, which is most commonly caused by three copies of chromosome 21, with the extra copy usually of maternal origin (Crow, 2000). However, we are not aware of any evidence supporting an excess of germline chromosomal abnormalities in childhood carcinoma cases. Other plausible biological hypotheses for a maternal age effect on disease risk in the offspring could involve epigenetic and mitochondrial mechanisms. For example, differences in gene expression have been noted between young and old oocytes (Hamatani *et al*, 2004; Steuerwald *et al*, 2007; Pan *et al*, 2008). The extent to which these differences are transmitted to offspring and could explain an increased childhood cancer risk of any subtype including carcinomas has not been well studied. However, transgenerational inheritance of epimutations is plausible with the report of a maternally inherited hypermethylated *MLH1* allele in a subject with early-onset colorectal carcinoma (Hitchins *et al*, 2007). With regard to mitochondrial mechanisms, it is plausible that maternally transmitted mitochondrial DNA mutations that increase with age could be linked to cancer in the offspring (Eichenlaub-Ritter *et al*, 2011).

Although based on small numbers, our data suggested stronger associations between older parental age and childhood carcinomas in younger individuals which would be consistent with the hypothesis of an underlying genetic predisposition caused by an increased frequency of germline mutations in older parents (Arver *et al*, 2000). However, it is also possible that advanced parental age could be a proxy for other non-genetic factors that are related to childhood cancer risk. Because our data set contained only a limited number of birth record variables, we could not assess whether the parental age associations were confounded by factors plausibly related to older parental age and the carcinomas under study such as vacation sun exposure for melanoma (Jen *et al*, 2009).

We observed a nearly two-fold increased risk of thyroid carcinoma in individuals born <37 weeks. To our knowledge, no previous study has examined the association between thyroid carcinoma and gestational age. Further study is necessary to determine if this finding could be the related to medical exposures that occur more frequently in pre-term infants, such as diagnostic radiation (Pizzo and Poplack, 2006; Lai and Bearer, 2008; Papadopoulou and Efthimiou, 2009).

The association between birth order and malignant melanoma has been examined in two studies conducted in Denmark and Sweden (Hemminki and Mutanen, 2001; Olesen *et al*, 2009). In ∼2.5 million Danes born from 1950 to 2002 that were followed for incidence cutaneous malignant melanoma diagnoses (*n*=1674) at all ages (i.e., children and adults), individuals with birth orders of five or higher had a significantly reduced risk of melanoma (IRR=0.24, 95% CI 0.06–1.01); however this result was based on only two cases in the highest birth order category (Olesen *et al*, 2009). In Sweden, in 1.38 million individuals <55 years who were born during 1958–1996 and followed for melanoma diagnoses (*n*=1101), a nonsignificant reduction in risk of 5% was observed per one unit increase in birth order (Hemminki and Mutanen, 2001). Further study is necessary to evaluate the role of birth order in malignant melanoma.

A strength of our study is that it is large and population based with birth characteristic data obtained before diagnosis. To our knowledge, this is the first study to specifically address the relationship between birth characteristics and paediatric carcinomas. Our study also has limitations. Although the accuracy of some characteristics may not be optimal, the variables that we used generally have high validity (Northam and Knapp, 2006) and any misclassification should be non-differential with regard to case *vs* control status. Because childhood carcinomas are rare, our statistical power to detect weak or modest associations was limited and thus our results should be considered exploratory. We also did not have follow-up of our control population, which could have resulted in missed cases. For example, if a control moved out of state and developed a childhood carcinoma, he/she would have not been included as a case in our data set. However, because childhood carcinomas are very rare it is unlikely that any controls developed this type of cancer; therefore it is also unlikely that this type of bias affected our results. Missing data could have biased the results if those with complete data were systematically different from those with missing data. The percentage of cases and controls with missing data on the variables included in this analysis was similar and generally <10% (with the exception of paternal age at 11%) making it seem unlikely that missing data had a strong influence on our results. Finally, it is possible that chance variation could be an explanation for our results.

In summary, we found that our results for childhood carcinomas reflected the same patterns seen for adults of a positive association between white race and melanoma. We found no evidence for an association between most other birth characteristics and childhood carcinomas with the possible exception of a relationship between birth order and melanoma, low gestational age and thyroid carcinoma, and a parental age effect for childhood carcinomas overall, and melanoma and thyroid carcinoma specifically.

## Figures and Tables

**Table 1:** Characteristics of childhood carcinoma cases in the pooled data set

	**Total (*n*=475)**	**Thyroid (*n*=159)**	**Malignant melanoma (*n*=126)**	**Hepatocellular (*n*=41)**	**Adrenocortical (*n*=21)**	**Renal cell (*n*=16)**	**Nasopharyngeal (*n*=13)**	**Gonadal (*n*=9)**	**Non-melanoma skin (*n*=5)**	**Others and unspecified (*n*=85)** a
*Diagnosis age (%)*										
0–4	17.7	4.4	27.0	41.5	66.7	0	0	11.1	0	12.9
5–9	20.6	20.1	17.5	24.4	23.8	37.5	23.1	11.1	60.0	18.8
10–14	61.7	75.5	55.6	34.2	9.5	62.5	76.9	77.8	40.0	68.2
Female (%)	55.0	74.0	41.0	39.0	67.0	44.0	46.0	100	40.0	44.0
Non-white (%)^b^	12.6	7.8	4.1	22.5	19.1	44.0	45.5	12.5	0	17.7

a 91% (*n*=77) were found in other tissue sites and 9% (*n*=8) were classified as having an unknown primary site.

b A total of 15 cases (6 thyroid carcinomas, 5 malignant melanomas, 1 hepatocellular carcinoma, 2 nasopharyngeal carcinomas, and 1 gonadal carcinoma) had missing data on maternal race.

**Table 2:** Associations between birth characteristics and any childhood carcinoma, thyroid carcinomas, and malignant melanomas diagnosed from 0–14 years

		**All carcinomas**				**Thyroid carcinomas**				**Malignant melanoma**			
	**Controls no. (%)**	**Cases no. (%)**	**OR**	**95% CI**		**Cases no. (%)**	**OR**	**95% CI**		**Cases no. (%)**	**OR**	**95% CI**	
*Maternal race* a,b													
White	45 671 (85)	371 (87)	0.98	0.74	1.31	130 (92)	1.54	0.82	2.88	109 (96)	3.27	1.33	8.06
Other	8057 (15)	56 (13)	1.0	Reference		11 (8)	1.0	Reference		5 (4)	1.0	Reference	
													
*Maternal age (years)* c, b													
Per 5-year increase	53 728 (100)	427 (100)	1.09	0.99	1.20	141 (100)	1.15	0.97	1.37	114 (100)	1.20	1.00	1.44
<25^c^	19 608 (36)	162 (38)	1.0	Reference		53 (38)	1.0	Reference		32 (28)	1.0	Reference	
25–29	17 174 (32)	140 (33)	1.08	0.85	1.37	47 (33)	1.06	0.71	1.60	44 (39)	1.67	1.05	2.67
30–34	11 769 (22)	90 (21)	1.17	0.89	1.54	25 (18)	1.00	0.60	1.65	29 (25)	1.85	1.09	3.13
⩾35	5177 (10)	35 (8)	1.25	0.85	1.85	16 (11)	1.88	1.02	3.48	9 (8)	1.62	0.75	3.51
													
*Paternal age* (*years)*c,^d^													
Per 5-year increase	48 000 (100)	380 (100)	1.10	1.01	1.20	130 (100)	1.16	1.01	1.33	107 (100)	1.13	0.96	1.32
<25	10 112 (19)	73 (19)	1.0	Reference		22 (17)	1.0	Reference		21 (20)	1.0	Reference	
25–29	14 698 (27)	124 (33)	1.19	0.88	1.59	45 (35)	1.39	0.83	2.33	33 (31)	1.09	0.63	1.91
30–34	13 287 (25)	109 (29)	1.31	0.96	1.79	36 (28)	1.44	0.83	2.49	34 (32)	1.40	0.80	2.45
⩾35	9903 (21)	74 (19)	1.36	0.96	1.91	27 (21)	1.68	0.93	3.05	19 (18)	1.20	0.63	2.28
													
*Gestational age (weeks)* a, b													
<37	4266 (8)	45 (11)	1.27	0.89	1.82	18 (13)	1.87	1.07	3.27	10 (9)	1.35	0.65	2.79
37–42	45 997 (86)	353 (83)	1.0	Reference		115 (82)	1.0	Reference		97 (85)	1.0	Reference	
>42	3465 (6)	29 (7)	1.05	0.71	1.53	8 (6)	0.93	0.45	1.91	7 (6)	0.93	0.43	2.00
													
*Plurality* a, b													
Multiple	1203 (2)	6 (1)	0.55	0.24	1.28	3 (2)	0.89	0.27	2.98	1 (1)	0.43	0.06	3.21
Single	52 525 (98)	421 (99)	1.0	Reference		138 (98)	1.0	Reference		113 (99)	1.0	Reference	
													
*Birth weight (g)* a, b													
Per 500 g	53 728 (100)	427 (100)	0.99	0.90	1.09	141 (100)	1.02	0.87	1.20	114 (100)	1.02	0.85	1.23
<2500	2911 (5)	32 (7)	1.27	0.83	1.95	9 (6)	0.76	0.35	1.65	5 (4)	0.82	0.30	2.26
2500–4000	43 989 (82)	346 (81)	1.0	Reference		123 (87)	1.0	Reference		90 (79)	1.0	Reference	
>4000	6828 (13)	49 (11)	1.02	0.75	1.38	9 (6)	0.59	0.30	1.17	19 (17)	1.31	0.80	2.17
													
*Birth order* a, b													
1	22 008 (41)	177 (41)	1.0	Reference		60 (43)	1.0	Reference		47 (41)	1.0	Reference	
2	17 543 (33)	142 (33)	0.98	0.78	1.23	45 (32)	0.89	0.59	1.32	42 (37)	1.01	0.66	1.55
3	8661 (16)	67 (16)	0.91	0.68	1.22	20 (14)	0.78	0.46	1.32	18 (16)	0.85	0.48	1.49
⩾4	5516 (10)	41 (10)	0.83	0.58	1.20	16 (11)	0.91	0.50	1.67	7 (6)	0.49	0.21	1.12

Abbreviations: CI=confidence interval; OR=odds ratio.

a Adjusted for state (CA, WA, MN, NY, TX), birth year category (1970–85, 1986–89, 1990–93, 1994–2004), maternal race (white, other), sex (male, female), maternal age as a continuous variable, gestational age (<37, 37–42, >42 weeks), plurality (multiple, single), birth weight (<2500, 2500–4000, >4000 g), and birth order (1, 2, 3, ⩾4).

b 4238 controls and 48 total carcinoma cases (*n*=18 thyroid carcinoma and 12 malignant melanoma cases) were excluded due to missing data on one or more covariates.

c Adjusted for state (CA, WA, MN, NY, TX), birth year category (1970–85, 1986–89, 1990–93, 1994–2004), maternal race (white, other), sex (male, female), gestational age (37–42, <37, >42 weeks), plurality (multiple, single), birth weight (<2500, 2500–4000, >4000 g), and birth order (1, 2, 3, ⩾4).

d 9966 controls and 95 total carcinoma cases (29 thyroid carcinoma and 19 malignant melanoma cases) were excluded due to missing data on one or more covariates.
