# Supplementary material for: Birth characteristics and childhood carcinomas
Source: Br J Cancer. 2011 Sep 13;105(9):1396–401. doi: 10.1038/bjc.2011.359 (PMC3241539; doi:10.1038/bjc.2011.359)
Supplement: Supplementary Table 1 [file bjc2011359x1.doc]

**Supplementary Table 1.** Associations between parental age and childhood carcinomas by age group and sex.

|  | **Maternal age** | | | **Paternal age** | | |
| --- | --- | --- | --- | --- | --- | --- |
| **Variable** | **Number of**  **controls/cases** | **OR^c^** | **95% CI** | **Number of controls/cases** | **OR^c^** | **95% CI** |
| *Age group* |  |  |  |  |  |  |
| **All carcinomas** |  |  |  |  |  |  |
| <11 | 209/50,550 | 1.10 | 0.97-1.26 | 183/44907 | 1.12 | 1.00-1.26 |
| >11^a^ | 218/33134 | 1.07 | 0.94-1.23 | 197/29492 | 1.09 | 0.96-1.22 |
|  |  |  |  |  |  |  |
| **Thyroid carcinomas** |  |  |  |  |  |  |
| <11 | 52/53728 | 1.17 | 0.89-1.53 | 49/48000 | 1.28 | 1.03-1.59 |
| >11^a^ | 89/33134 | 1.15 | 0.93-1.42 | 81/29492 | 1.09 | 0.91-1.31 |
|  |  |  |  |  |  |  |
| **Melanoma** |  |  |  |  |  |  |
| <11 | 63/53728 | 1.23 | 0.97-1.55 | 57/48000 | 1.14 | 0.93-1.41 |
| >11^a^ | 51/33134 | 1.17 | 0.88-1.55 | 50/29492 | 1.10 | 0.87-1.40 |
|  |  |  |  |  |  |  |
| *Sex^b^* |  |  |  |  |  |  |
| **All carcinomas** |  |  |  |  |  |  |
| Male | 194/28343 | 1.08 | 0.93-1.24 | 170/25352 | 1.05 | 0.93-1.20 |
| Female | 233/25385 | 1.10 | 0.97-1.25 | 210/22648 | 1.14 | 1.02-1.28 |
|  |  |  |  |  |  |  |
| **Thyroid carcinomas** |  |  |  |  |  |  |
| Male | 37/28343 | 1.08 | 0.78-1.51 | 36/25352 | 1.11 | 0.85-1.47 |
| Female | 104/25385 | 1.18 | 0.98-1.44 | 94/22648 | 1.18 | 1.00-1.39 |
|  |  |  |  |  |  |  |
| **Melanoma** |  |  |  |  |  |  |
| Male | 69/28343 | 1.17 | 0.93-1.48 | 63/25352 | 1.10 | 0.90-1.35 |
| Female | 45/25385 | 1.27 | 0.95-1.69 | 44/22648 | 1.17 | 0.92-1.49 |

^a^Excluded subjects born after 1993 and California subjects due to no cases diagnosed over age 11 years.

^b^P-values for logistic regression models that included interaction terms between sex and the respective parental age variable were not statistically significant.

^c^ Adjusted for state (CA, WA, MN, NY, TX), birth year category (1970-85, 1986-89, 1990-93, 1994-2004), maternal race (white, other), sex (male, female), gestational age (37-42, <37, >42 weeks), plurality (multiple, single), birth weight (<2500, 2500-4000, >4000 grams), and birth order (1, 2, 3, >4). ORs for sex specific estimates of associations between parental age and childhood carcinomas were also adjusted for maternal age as a continuous variable.
